# Various tomato cultivars display contrasting morphological and molecular responses to a chronic heat stress

**DOI:** 10.3389/fpls.2023.1278608

**Published:** 2023-10-25

**Authors:** N. Bollier, R. Micol-Ponce, A. Dakdaki, E. Maza, M. Zouine, A. Djari, M. Bouzayen, C. Chevalier, F. Delmas, N. Gonzalez, M. Hernould

**Affiliations:** ^1^ INRAE, Université de Bordeaux, BFP, Bordeaux, France; ^2^ Laboratoire de Recherche en Sciences Végétales, Université de Toulouse, CNRS, UPS, Toulouse INP, Toulouse, France

**Keywords:** heat stress (HS), pollen development, HS transcriptional response, tomato, flower development

## Abstract

Climate change is one of the biggest threats that human society currently needs to face. Heat waves associated with global warming negatively affect plant growth and development and will increase in intensity and frequency in the coming years. Tomato is one of the most produced and consumed fruit in the world but remarkable yield losses occur every year due to the sensitivity of many cultivars to heat stress (HS). New insights into how tomato plants are responding to HS will contribute to the development of cultivars with high yields under harsh temperature conditions. In this study, the analysis of microsporogenesis and pollen germination rate of eleven tomato cultivars after exposure to a chronic HS revealed differences between genotypes. Pollen development was either delayed and/or desynchronized by HS depending on the cultivar considered. In addition, except for two, pollen germination was abolished by HS in all cultivars. The transcriptome of floral buds at two developmental stages (tetrad and pollen floral buds) of five cultivars revealed common and specific molecular responses implemented by tomato cultivars to cope with chronic HS. These data provide valuable insights into the diversity of the genetic response of floral buds from different cultivars to HS and may contribute to the development of future climate resilient tomato varieties.

## 1 Introduction

Population growth and global warming are two of the major issues that humanity will have to face in the next decades (IPCC, 2014). Based on current climatic models, experts commonly anticipate that rising temperatures will cause substantial yield losses in the future affecting major plant-food sources (Tripathi et al., 2016). Global warming has already a significant impact on crop production and agricultural practices. During the past years, many regions of the world underwent extreme heat waves causing severe damages to ecosystems, human society and crop production (Stillman, 2019). The frequency and intensity of these heat waves will increase in many regions of the globe leading to major losses in agricultural yield (Lau and Nath, 2012; Molina et al., 2020).

Plant growth and development can be strongly altered by high temperatures, the most heat-susceptible phases being early seedling and reproductive phases. Heat-stress (HS) can impair essential cellular, physiological and developmental processes, including genome integrity, photosynthesis, flower development or pollen development and viability as reported for a number of plant species (Han et al., 2021; Moore et al., 2021). Detrimental effects of heat on pollen production have been described in various plant taxa, including *Arabidopsis thaliana* (Arabidopsis), *Solanum lycopersicum* (tomato) and *Oryza sativa* (rice) and for different developmental stages such as meiosis, microspore development and pollen maturation (Raja et al., 2019). Pollen development is known to be one of the most temperature-sensitive process in plant life cycle (Zinn et al., 2010), and alteration of pollen meiosis, particularly at the tetrad stage, has been reported in different species such as Arabidopsis, maize or cotton (Begcy et al., 2019; De Storme and Geelen, 2020; De Jaeger-Braet et al., 2021; Masoomi-Aladizgeh et al., 2021; Ning et al., 2021) and impaired pollen development results in poor fertilization and reduced fruit and seed yield (Giorno et al., 2013; Müller and Rieu, 2016).

The cellular responses to HS have been studied in a variety of plant species and various organ and tissues revealing both common and specific responses. HS disturbs various cellular processes, resulting, for example, in the denaturation of biological molecules, such as proteins, lipids, and nucleic acids (Bohnert et al., 2006; Kotak et al., 2007; Essemine et al., 2010) and the disintegration of subcellular structures, including membranes and cytoskeleton networks (Savchenko et al., 2002; Saidi et al., 2011). HS affects genome integrity by triggering DNA damage through nucleotide modifications and single-strand or double-strand breaks (Kantidze et al., 2016) and it also alters chromatin architecture (Pecinka and Mittelsten Scheid, 2012). To cope with HS, plants have developed highly complex intracellular signaling systems involving hormones, Ca^2+^ and reactive oxygen species (ROS) (Wahid et al., 2007). For a long time, ROS were considered as a byproduct that impairs plant growth. However, ROS recently gained attention for their function as signaling molecules involved in response to environmental stresses like HS (Medina et al., 2021). In support to this idea, it has been shown that ROS-scavenging enzymes, involved in detoxification processes, are rapidly induced by HS (Suzuki and Mittler, 2006).

HS induces rapid transcriptional changes and the elucidation of the complex transcriptional regulatory networks involved in plant responses to HS is now well advanced (Ohama et al., 2017). That is, HS rapidly activates HS-responsive transcription factors, HSFs, which regulate the transcription of a wide range of target genes involved in signaling and metabolic pathways (von Koskull-Döring et al., 2007). Among the most notable responsive proteins, the Heat Shock Proteins (HSPs) are known to play essential roles in cellular protection through their action on protein misfolding or aggregation but also in protein translocation and degradation (Vierling, 1991; Schleiff and Becker, 2011). Transcriptomic profiling has identified several molecular pathways induced under elevated temperature conditions including photosynthesis, response to light or rRNA processing (Jayakodi et al., 2019). Interestingly, in addition to the classically induced HSPs, these studies suggested potential roles for several factors involved in epigenetic, post-transcriptional and post-translational regulation as well as in hormonal regulation. Deciphering the effects of HS at the transcriptome level greatly helped defining candidate genes potentially involved in mediating plant tolerance to HS and several attempts aiming to improve thermotolerance by knocking out or overexpressing these candidate genes have been reported in different plant species including Arabidopsis, tomato, rice and *Glycine max* (soybean) (Zhu et al., 2006; Yokotani et al., 2008; Xin et al., 2010; Wu et al., 2012; Li et al., 2013; Shen et al., 2015; Xue et al., 2015; Wan et al., 2016). It is however, likely that the activation of two or more independent -but mutually complementary- pathways would improve more effectively thermotolerance in crops species. In this regard, new pathways need to be discovered in order to pave the way towards the generation of highly tolerant genotypes using the newly identified candidates for gene stacking.

HS response has been extensively investigated in plants in the past two decades. Most studies have been carried out applying very high temperatures (45°C to 50°C) for a short period of time (from 30 minutes to 3 hours) on the whole plant, or focused on specific developmental stages or organs (Qu et al., 2013; Driedonks et al., 2016; Janni et al., 2020). Strikingly so far, quite a few studies have been carried out that simulate heat wave conditions as defined by the STAtistical and Regional dynamical Downscaling of EXtremes for European regions (STARDEX) project: at least 5 consecutive days with 5 degrees anomaly with respect to mean temperature in summer (Jagadish et al., 2021). Similarly, only few studies have investigated the response of plants to chronic HS, corresponding to longer periods of heat (Sato et al., 2000). Because the effect of short-term heat treatments is unlikely to completely reproduce what happens during long term HS, studying the impact of long periods of heat on plants is essential to eventually select plant cultivars with increased heat tolerance.

Tomato is one of the most produced and consumed fruits in the world. However, tomato producers face dramatic yield losses in very warm summers, due to poor fruit setting resulting in decreased fruit number and small fruits of low quality (Adams et al., 2001). In recent years, several transcriptomic, metabolomic, proteomic or lipidomic analyses were performed to study the response of tomato plants to HS (Pressman et al., 2002; Spicher et al., 2016; Paupière et al., 2017; Almeida et al., 2021). Studies focusing on floral buds revealed that short-term HS induces the expression of *HSF* genes, *HSP* genes, ROS scavenger genes and genes involved in the control of sugar levels (Frank et al., 2009). However, most of these studies are limited to one tomato genotype and, more importantly, the short-term treatment is unlikely to completely reproduce the molecular responses to chronic stress.

Here, we aimed at identifying the common and/or specific molecular responses to a chronic HS in tomato floral buds of various tomato cultivars. Seeds from 11 modern tomato cultivars were obtained from different seed companies, and plants were grown and subjected to a chronic HS of 3 weeks, before evaluating the pollen germination rate. Among these 11 cultivars, 2 and 3 cultivars with high and low pollen germination rate under HS respectively, were selected for subsequent genome-wide transcriptomic profiling analysis of their floral buds to identify the molecular pathways regulated during a chronic HS. These analyses highlighted the different genetic responses that tomato cultivars set-up for facing chronic HS.

## 2 Materials and methods

### 2.1 Plant material and growth

Plant material consisted of eleven different tomato cultivars: Brioso (Rijk Zwaan), Clodano (Syngenta™), Docet (Monsanto™), DRK7024 (De Ruiter™), JAG8810 (Bayer™), M82, Marbonne (Gautier Semences), Moneymaker, Rebelski (De Ruiter™), Sassari (Rijk Zwaan) and West Virginia 106 (WVA106). This set of cultivars was selected in order to cover some of the tomato diversity in terms of i) fruit morphology: small (WVA106, Sassari, Brioso, M82), medium (Docet, Moneymaker, Clodano, JAG8810), and large size (Rebelski, Marbonne, DRK7024) (**Figure S2**); ii) growth pattern: determinate (JAG8810, M82) *versus* indeterminate growth (DRK7024, WVA106, Brioso, Marbonne, Sassari, Moneymaker, Rebelski); and iii) market suitability: fresh market (Sassari, Brioso, M82, Clodano, Moneymaker) and processing tomatoes (JAG8810, DOCET). In non-stress (NS) conditions, plants were cultivated in a greenhouse with a photoperiod of 16h/8h with a mean temperature of 24°C during the day and a mean temperature of 18°C during the night. For HS treatments, the plants at bolting stage have been submitted during 3 weeks to a mean temperature of 35°C during the day (16h) and 25°C during the night (8h) using the main and two auxiliary heaters and the temperature has been monitored all along the HS (**Figure S1A**). In both conditions, the hygrometry was around 55% all day and plants had a daily watering.

### 2.2 Pollen developmental stage analysis

To analyze pollen development, 3-5 floral buds for each different size were dipped in Carnoy’s fixative solution (60% (v/v) ethanol, 30% (v/v) chloroform, 10% (v/v) acetic acid). Then, the anthers from the flower buds were dissected in acetocarmine staining solution (1% (p/v) carmine 40, 0.5% (p/v) ferric chloride, 45% (v/v) glacial acetic acid) using gauge needles under a stereomicroscope and squashed between a slide glass and a coverslip to release the male reproductive cells as described by (Puchtler et al., 1968). Pollen developmental stages were observed using a bright field microscope (Zeiss, Axioplan) and photographed with a CCD camera (Motic 3 megapixels).

### 2.3 Pollen germination assays

Pollen grains from 3 to 5 tomato flowers at anthesis stage were sprayed on the top of pollen germination medium (18% sucrose, 0.01% Boric acid, 1mM CaCl_2_, 1mM Ca(NO_3_)_2_, 1mM MgSO_4_, 0.5% agar; pH=7). Each experiment was repeated 3 times. After 16h of incubation at 25°C in the dark, the preparation was observed using a stereomicroscope Olympus SXZ16 and photographed using a camera (Motic 10 megapixels). The pollen germination rate expressed as a percentage was determined by dividing the number of germinated pollens corresponding to those emitting a pollen tube by the total number of pollen grains.

### 2.4 Genome wide expression profiling

To prepare RNA samples for the subsequent RNAseq analysis, each tomato cultivar was grown under NS or HS conditions [3 weeks at 35°C during the day (16h) and 25°C during the night (8h)]. Based on the histological analysis, floral buds were harvested at the tetrad stage (tetrad floral bud, TFB) and at the pollen mature stage (pollen floral bud, PFB) based on their size (**Table S1**). Five to ten floral buds for each stage were sampled before and 20 days after the start of the HS from 20-25 plants per genotype for the RNAseq analysis (**Figure S1B**). Total RNA was isolated from 200 and 500 mg of floral buds at different developmental stages using TRIzol Reagent (Life Technologies). Total RNA extract was purified with the Qiagen RNAeasy mini kit RNA. After DNase treatment (DNA-free Kit, Life Technologies), the total RNA quantity and quality (RNA integrity number, RIN) were evaluated using an Agilent 2100 Bioanalyzer (Agilent Technologies). Only RNA extracts with a RIN of 10 were used for sequencing. The RNA libraries were constructed as described in the Illumina TruSeq Stranded mRNA guide. mRNAs were sequenced in a HiSeq 3000 sequencing system with 2 × 125 bp paired-end sequences (Illumina HiSeq SBS Kit v4) by the Genotoul bioinformatics platform, Toulouse (http://bioinfo.genotoul.fr/index.php).

Three biological replicates per plant and per stage were harvested for NS and HS conditions, resulting in 60 samples (5 cultivars × 2 conditions × 2 developmental stages × 3 biological replicates). To obtain the genome-wide expression profiling, RNA samples were subjected to next-generation sequencing.

For each RNA sample, more than 20 million of paired-end reads (10 million fragments) were generated, using an Illumina Hiseq 3000 platform. More than 95% of total clean reads were mapped against the SL3.0 version of the reference tomato genome for read mapping (ftp://ftp.solgenomics.net/genomes/Solanum_lycopersicum/Heinz1706/assembly/build_3.00/) and almost 90% of the mapped reads matched a feature on the related gene model.

### 2.5 Bioinformatic analyses

Statistical analyses and graphs have been performed with the R software and homemade scripts. The DE analysis has been carried out with the DESeq2 R-package (Love et al., 2014). Some analyses, such as the PCA analysis, were carried out with functions contained in the DESeq2 package. Reads were checked using fastQC, cleaned using trimGalore, and mapped using Star, a spliced aware mapper software on the new tomato genome SLmic1.0 generated in the frame of TOMGEM project (http://tomatogenome.gbfwebtools.fr/). The mapping was guided using the gene model annotation version 1.1. FeatureCount was then used to calculate the read counts for each gene from each mapping file. A normalization step was performed in order to obtain comparable expression values between conditions and between genes. For this purpose, our pipeline takes into account the relative size of studied transcriptomes, the library sizes and the gene lengths, as described in Maza et al. (2013); Maza (2016). After data normalization using DESeq2 package, a PCA analysis has been conducted with expression data in all conditions and replicates, in order to check global sample variability.

Genome-wide expression data have been analyzed in order to identify genes differentially expressed between different conditions that have been tested. The “Relative Log Expression” normalization (RLE) implemented in the DESeq2 package has been used as normalization method. To highlight DEGs, the genes exhibiting an adjusted *p-value* < 0.05 and a log2 fold-change < -1 or > +1 have been selected. To eliminate very lowly expressed genes, the up-regulated DEGs with a count value < 10 in HS condition and the down-regulated DEGs with a count value < 10 in NS condition were filtered out.

To calculate the intersections of DEGs lists within the different conditions, Venn diagrams corresponding to textual outputs were generated by using the Venn diagram tools at http://bioinformatics.psb.ugent.be/webtools/Venn/BAR Website at http://bar.utoronto.ca/or at https://www.biovenn.nl/index.php (Hulsen et al., 2008) for area-proportional Venn diagrams. The TomExpress web site, at http://tomexpress.toulouse.inra.fr/query, was used to extract gene expression data obtained from previous genome-wide expression analyses (Zouine et al., 2017).

The GO enrichment analysis has been carried out on PLAZA 5.0 (Van Bel et al., 2022) using the Plaza workbench, with a significance threshold of 0.05 and without any data filter.

## 3 Results

### 3.1 Morphological analyses of flower development and variability in pollen germination of tomato cultivars under chronic HS

**Figure 1:**
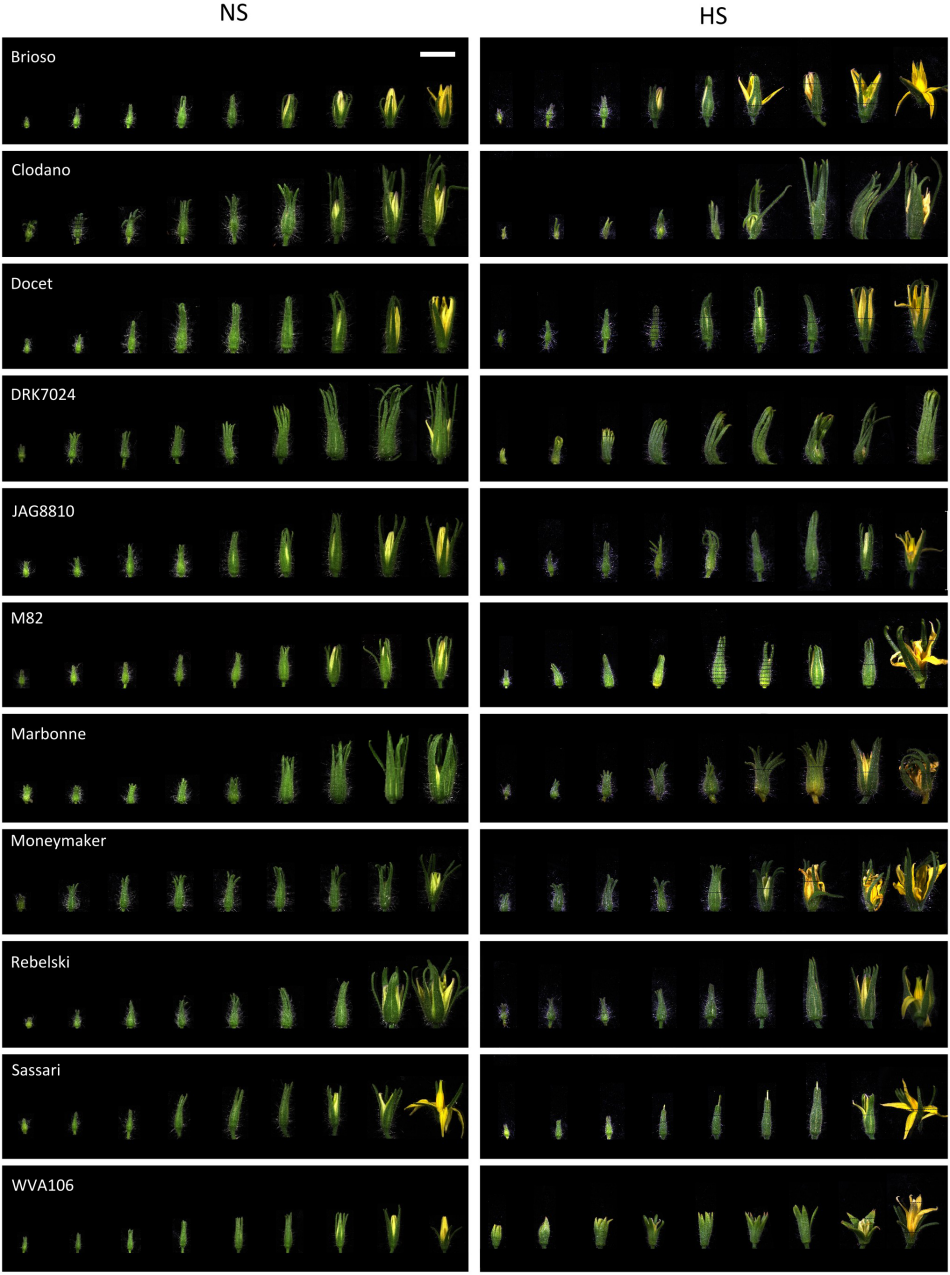
Floral bud development under heat stress (HS) and non-stress (NS) conditions in the 11 studied tomato cultivars. For each cultivar, floral buds are ordered according to their size in each condition showing the effect of the HS at different developmental stages. Bar, 1 cm (applies to all panels).

To assess phenotypic differences in response to HS of various tomato genotypes, we examined the behavior of eleven tomato cultivars corresponding to ten commercial varieties and the cherry tomato West Virginia 106 (WVA106) cultivar. Given that floral development is critical for tomato yield, we focused our analysis on the morphological and cytological changes occurring during this process under HS conditions. Under NS conditions, the 11 selected cultivars displayed variability in size and morphology of floral buds during development (**Figure 1**). Floral bud size distribution was not statistically different under NS and HS conditions (**Figure S3**), but clear morphological alterations were observed (**Figure 1**). In particular, only the flowers of the Sassari cultivar exhibited stigma exertion under HS compared with the complete absence of stigma exertion under NS conditions. On the other hand, HS induced the appearance of curled petals in Clodano, Docet, M82, Marbonne and Moneymaker cultivars. In addition, HS caused the sepals and petals to open at an earlier developmental stage than in NS conditions in Moneymaker, Brioso and WVA106. In this latter cultivar, sepals displayed burned extremities due to HS. In conclusion, floral development in various tomato cultivars was affected differently under HS, suggesting that the response to HS may be, at least partially, dependent on the genetic background.

**Figure 2:**
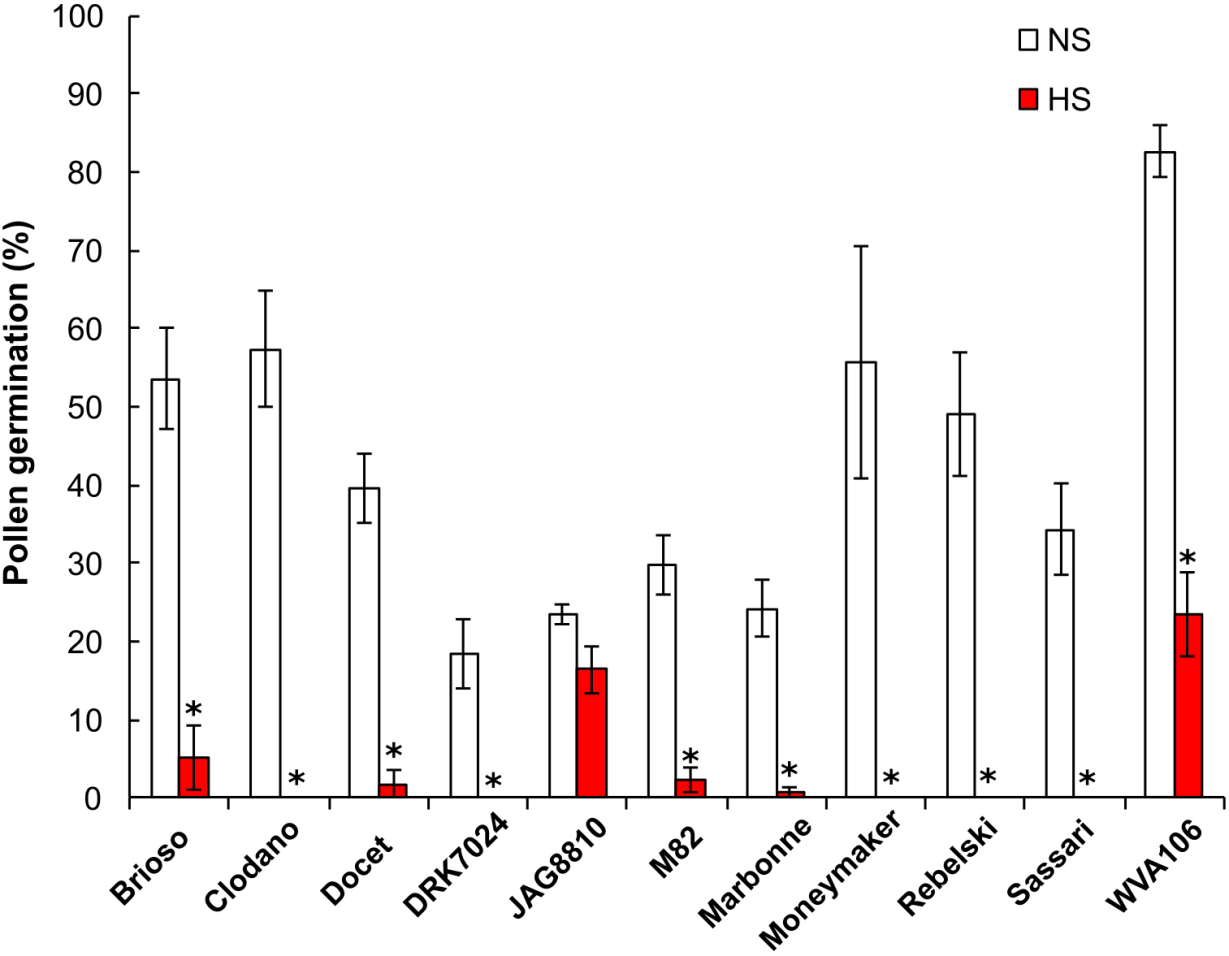
Pollen germination rate expressed as the percentage of germinating pollen grains (%) under non-stress (NS) and heat stress (HS) conditions in 11 tomato cultivars. *for ANOVA *p-value*<0.05.

Flower fertilization, and subsequent fruit set and seed production, can be impaired by poor pollen germination and pollen tube growth. We thus determined the percentage of pollen germination, used as a proxy for pollen potential for fertilization in the eleven tomato cultivars under NS and HS conditions (**Figure 2**). Under NS conditions, pollen germination was highly variable between the cultivars studied, with a maximum percentage of 80% for WVA106, and a minimum of 18.6% for DRK7024. Overall, the HS negatively impacted pollen germination in all cultivars, with a complete absence of germination in Clodano, DRK7024, Moneymaker, Rebelski and Sassari, indicating the extreme sensitivity to HS of the pollen in these cultivars (**Figure 2**). In Brioso, Docet, M82 and Marbonne cultivars, the pollen germination rate under HS was reduced respectively, by 10-, 21-, 12- and 33-fold, when compared to NS condition, indicating a high sensitivity to HS of these cultivars even though some germination capacity was maintained. Conversely, pollen germination in JAG8810 and WVA106 was only reduced by 1.4- and 3.5-fold respectively, suggesting a better tolerance to HS treatment in these cultivars. Taken together, these results indicated that pollen germination percentage is highly affected under HS conditions although the degree of sensitivity is highly genotype-dependent.

### 3.2 Cytological analyses of pollen development of tomato cultivars under chronic HS

**Figure 3:**
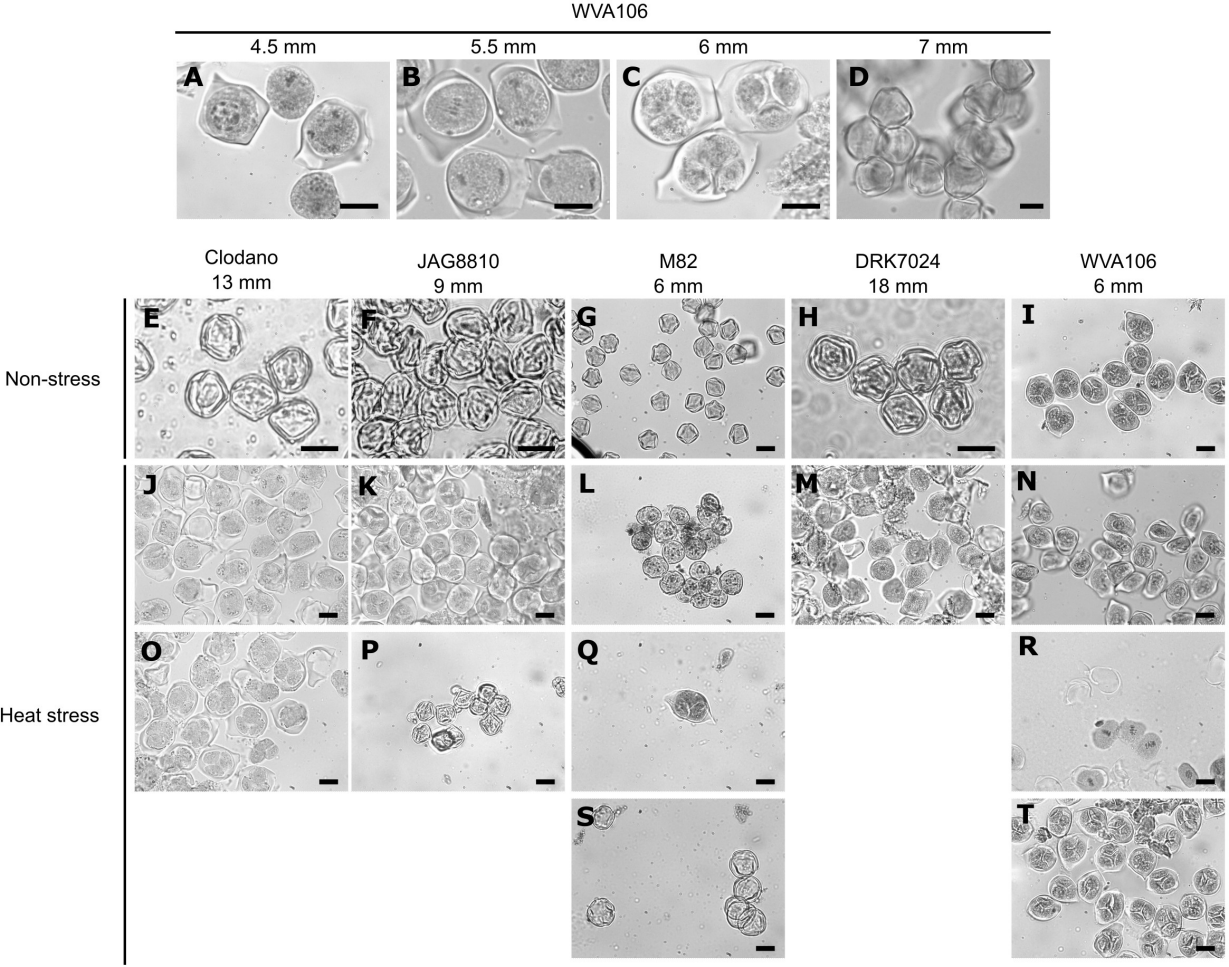
Histological analyses of pollen development in relation with bud size under non-stress (NS) and heat stress (HS) conditions in different tomato cultivars. Pollen developmental stages observed are the following: microspore mother cell **(A–M, N)**; Meiosis **(B, J, R)**; Tetrad **(C, I, K, O, Q, T)**; Mature pollen grains **(D–H, P, S)**. Bar, 20µm.

To determine if the effect of the chronic HS on pollen germination was related to a perturbation of pollen development, we selected 5 cultivars showing contrasting behaviors for pollen germination under HS: namely WVA106 and JAG8810 as tolerant cultivars, and Clodano, DRK7024 and M82 as sensitive cultivars. It has been described that under NS conditions, tomato floral bud size is generally synchronized with the developmental stage of the reproductive organs (Brukhin et al., 2003). Therefore, we determined first, under our NS growth conditions, the developmental stage of the male gametophyte in relation to floral bud size, using a histological approach. Four pollen developmental stages were considered because they were easily recognizable: Microspore Mother Cell (**Figure 3A**), present in 4.5 mm buds in WVA106; Meiosis (**Figure 3B**), in 5.5 mm buds in WVA106; Tetrad (**Figure 3C**) in 6 mm buds in WVA106 and Mature pollen grain (**Figure 3D**) in 7 mm buds in WVA106. Most of the time, only one pollen developmental stage was observed for a determined bud size under NS conditions, indicating the synchronization of floral bud and pollen development in the different cultivars studied. Indeed, Clodano, JAG8810, M82 and DRK7024 floral buds at 13 mm, 9 mm, 6 mm, and 18 mm respectively, only contained mature pollen grains (**Figures 3E–H**) and 6 mm floral buds in WVA106 only contained tetrads (**Figure 3I**). Under HS conditions, all cultivars, except DRK7024, showed several pollen developmental stages at these bud sizes (**Figures 3J–T**). For DRK7024, while only one pollen developmental stage was observed at a time, pollen development was delayed compared to NS condition as indicated by the observed microspore mother cell instead of mature pollen in 18 mm floral buds (**Figures 3H, M**).

**Figure 4:**
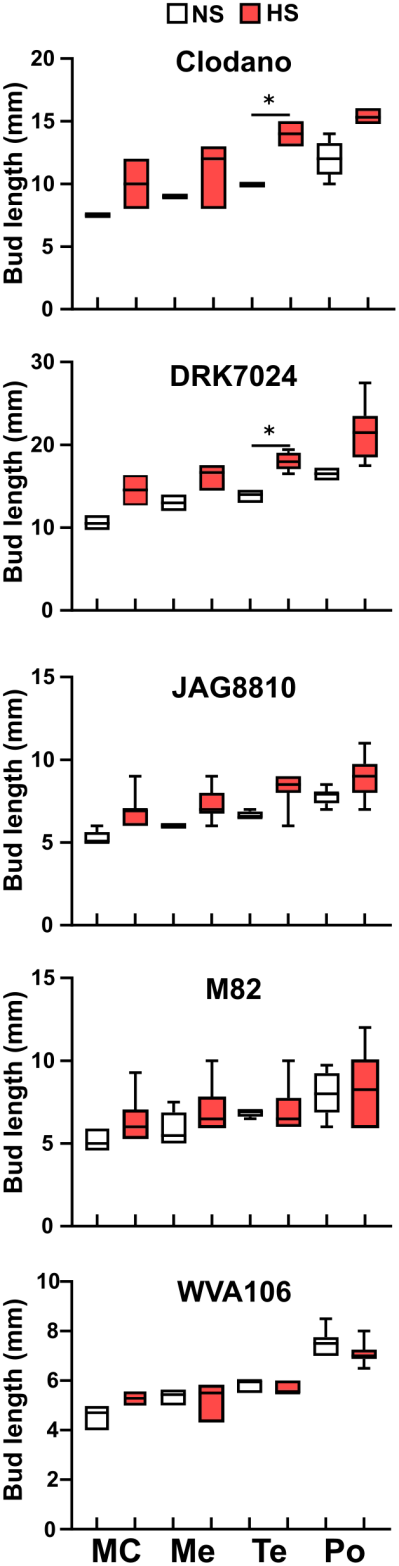
Size of floral buds for a defined pollen developmental stage in Clodano, DRK7024, JAG8810, M82, WVA106. MC, microspore mother cell; Me, Meiosis; Te, Tetrad; Po, Mature Pollen. * p-value<0.05 (Kruskal Wallis test).

These results were confirmed when reporting the size of the flower buds in relation to pollen developmental stage. In three cultivars (Clodano, DRK7024 and JAG8810), the stressed flower buds showed more size variability than in NS conditions for most pollen developmental stages and their length was significantly higher for Clodano and DRK7024 at the tetrad stage thus suggesting a delay in pollen development (**Figure 4**). Conversely, for a defined pollen developmental stage in WVA106 and M82, the floral bud size was not different between HS and NS indicating that pollen development was not delayed but rather desynchronized under HS condition in these two cultivars (**Figure 4**). Altogether, these results indicate that pollen development is either delayed (Clodano, DRK7024) and/or desynchronized (Clodano, JAG8810, M82, WVA106) by HS with a clear effect at the tetrad stage and regardless of the observed tolerance to the HS in terms of pollen germination capacity as determined in **Figure 2**.

### 3.3 General transcriptomic response to HS

To identify the molecular pathways differentially regulated in the floral buds of different tomato cultivars under HS, we carried out a genome-wide transcriptomic analysis by RNAseq on the five previously studied cultivars at two developmental stages: tetrad stage (tetrad floral bud, TFB) and pollen mature stage (pollen floral bud, PFB).

Following quality check and pre-processing of the sequence data, the reads were mapped against the SLmic1.0 tomato reference genome (gene model version 1.1). PCA of the RNA sequencing data showed that the samples clustered together according to both developmental stages and growth conditions factors explaining, respectively, about 60% and 10% of the whole variance (**Figure S4**). In TFB and PFB, the transcriptomic profiles corresponding to samples subjected to HS were clearly separated from the ones in NS conditions, indicating that the simulated chronic HS triggered a transcriptional response in all cultivars.

Differentially expressed genes (DEGs) in HS versus NS conditions were computed for each condition and for each developmental stage as described in materials and methods. The RNA-seq analysis yielded a total of 26017 expressed genes among which 3647 were significantly differentially expressed (adjusted p-value<0.05, 1<Log2FC< -1) across the five cultivars at the two stages and between the two growth conditions (**Table S2**). To analyze the global response to HS at each developmental stage independently of the cultivar considered, the down- or up-regulated genes from all cultivars were pooled and classified based on their GO annotation focusing on biological processes. In both PFB (1415 up and 1420 down, **Table S3**) and TFB (1369 up and 929 down, **Table S3**), most DEGs having enhanced or decreased expression in response to HS belonged to GO classes linked to response to stimulus, response to stress and response to abiotic stimulus (GO:0050896; GO:0006950 and GO:0009628 respectively) corresponding to 20 to 40% of the set of genes with an associated GO term in the studied list (**Table S4**). As expected, the categories “response to heat” (GO:0009408) and “cellular response to heat” (GO:0034605) were found enriched in the upregulated DEGs. Additionally, “response to reactive oxygen species” (ROS, GO:0000302), “response to oxygen-containing compound” (GO:1901700) and “cellular response to oxygen-containing compound” (GO:1901701) were enriched categories, containing around 15% of the PFB and TFB up-regulated genes. Interestingly, down-regulated genes corresponding to the GO class, term or category “sporopollenin biosynthetic process” (GO:0080110) were found enriched in PFB suggesting that pollen development might be affected. This global analysis thus reveals that the floral buds at both tetrad and pollen developmental stages were severely affected by the HS resulting in an oxidative stress.

**Figure 5:**
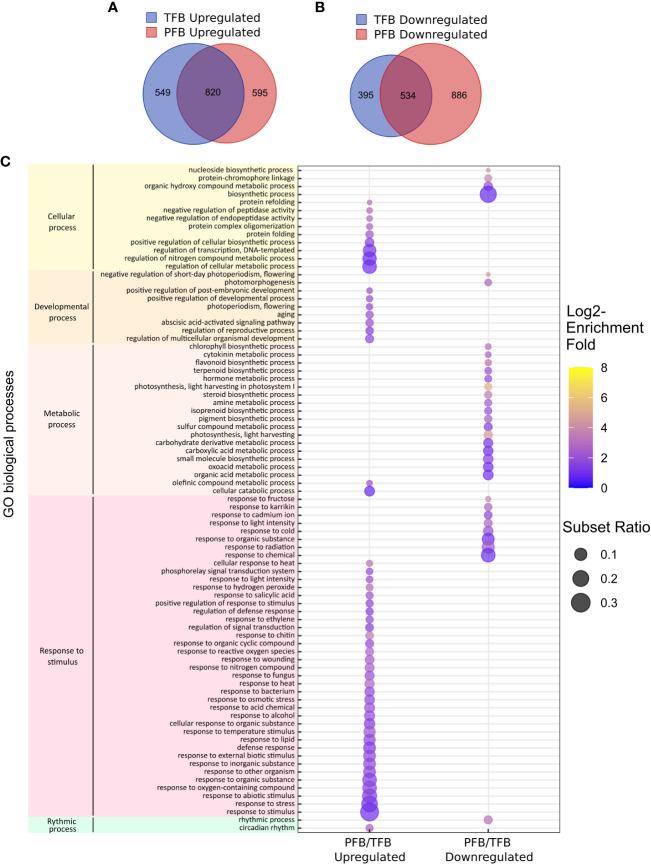
Global transcriptome response to HS in all tomato cultivars. **(A, B)** Venn diagram presenting the overlap of DEGs between upregulated **(A)** and downregulated **(B)** genes between TFB and PFB stages in all cultivars. **(C)** Gene ontology (GO) enrichment analyses of the common DEGs between PFB and TFB. The dot size is representative of the number of DEGs associated with the process and the fold enrichment is according to a heat map (dot color).

We then analyzed the DEGs commonly up- or down-regulated between PFB and TFB stages. 820 out of 1964 (42%) and 534 out of 1815 (29%) DEGs were found to be commonly up-regulated and down-regulated, respectively in PFB and TFB samples, highlighting the response similarity to HS independently of the developmental stage (**Figures 5A, B**). Most DEGs with enhanced expression in response to HS belonged to GO classes linked to response to stimulus such as response to heat (GO:0009408, GO:0034605) including 16 HSPs, response to ROS (GO:0000302 and GO:1901700) including ACC-oxidases, ascorbate peroxidase and catalases, and cellular processes such as protein folding (GO:0006457 and GO:0042026) (**Figure 5C**; **Table S5**). For the down-regulated DEGs, the GO enrichment analysis revealed mainly classes related to metabolic processes such as photosynthesis (GO:0009765). These results indicated that the global tomato transcriptome response at both TFB and PFB stages is largely common and suggested that both tissues undergo inhibition of photosynthetic processes.

### 3.4 Cultivar specific and common transcriptomic response to HS

**Figure 6:**
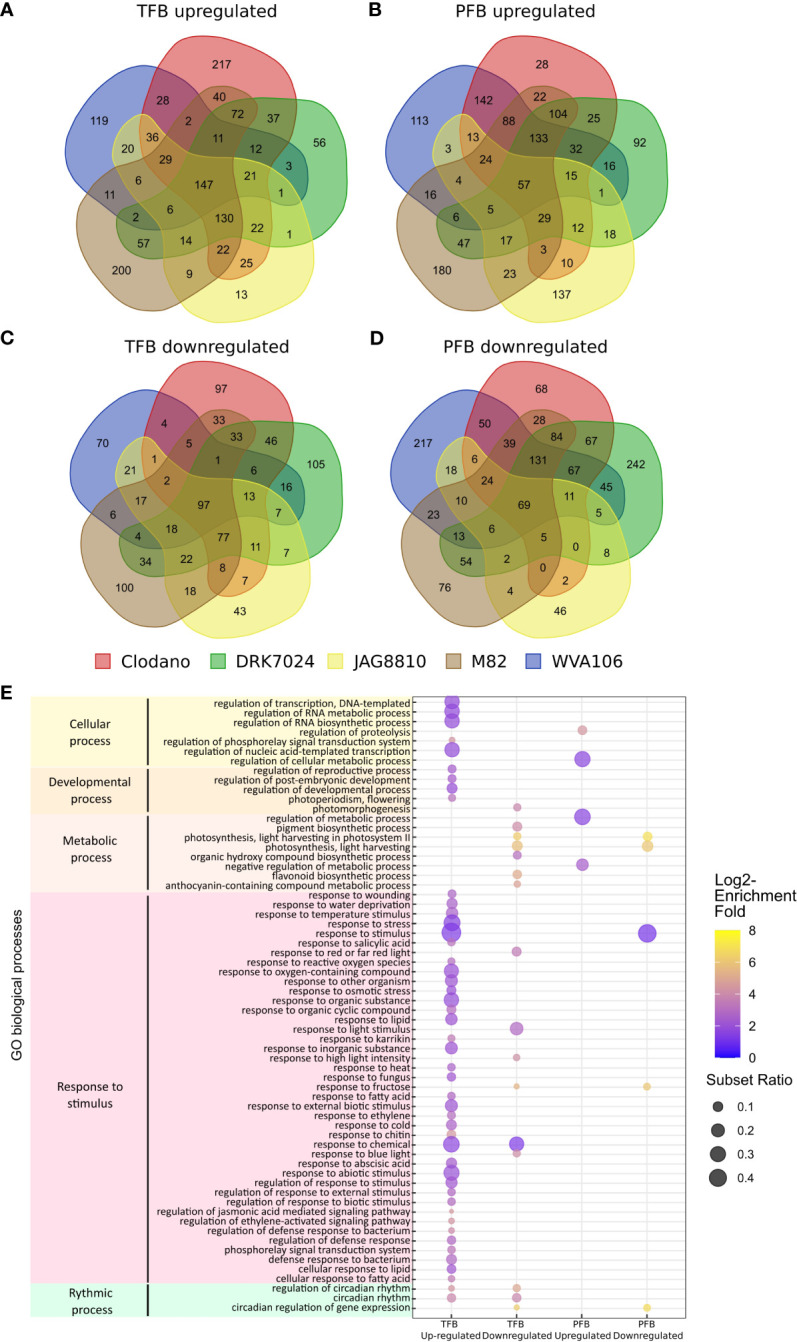
Common transcriptome response to HS between all cultivars. **(A–D)** Venn diagram presenting the overlap of DEGs between all cultivars: upregulated genes in TFB **(A)** and in PFB **(B)**; downregulated genes in TFB **(C)** and in PFB **(D)**. **(E)** Gene ontology (GO) enrichment analyses of the common DEGs between all cultivars. The dot size is representative of the number of DEGs associated with the process and the fold enrichment is according to a heat map (dot color).

To further evaluate the common and specific responses to HS between cultivars, we identified the DEGs in the different tomato cultivars under HS condition. At the TFB stage, we found 872 and 742 DEGs for JAG8810 and WVA106, respectively, considered as pollen tolerant cultivars *i.e.* cultivars for which the pollen germination rate was lowly affected under HS, and 1233, 1089 and 1293 DEGs for M82, DRK7024 and Clodano, respectively, considered as pollen sensitive cultivars *i.e.* cultivar for which the pollen germination rate was highly reduced under HS (**Table S3**). At the PFB stage 587 and 1402 DEGs were found for JAG8810 and WVA106 respectively, and 1327, 1418 and 1388 for M82, DRK7024 and Clodano respectively. We then compared the DEGs found in the five cultivars at each developmental stage. The Venn diagram of the up-regulated genes in TFB showed that 147 genes were common between all cultivars which represent between 17% (for Clodano) and 32% (for WVA106) of the up-regulated DEGs (**Figure 6A**; **Table S6**). In PFB, 57 genes were found common between the five cultivars representing between 7% (for M82) and 15% (for JAG8810) of the up-regulated DEGs (**Figure 6B**; **Table S6**). Interestingly only 13 out of 502 genes were found specifically up-regulated in JAG8810 indicating that the response at this stage in this cultivar is almost completely shared by the others. Concerning the down-regulated DEGs, 97 genes (representing between 20% for DRK7024 and 34% for WVA106) and 69 genes (representing between 9% for DRK7024 and 32% for JAG8810) were commonly down-regulated in TFB and PFB, respectively, between all cultivars (**Figures 6C, D**; **Table S6**).

The 147 commonly up-regulated DEGs identified in TFB were mainly involved in response to stimulus (GO:0050896) with 49% of the set of the genes with an associated GO term in the studied list (**Figure 6E**; **Table S7**). As expected, the GO term “response to heat” (GO:0009408) and “response to oxygen-containing compound” (GO:1901700) were found enriched. Similarly, GO terms corresponding to hormonal responses including abscisic acid (GO:0009737), salicylic acid (GO:0009751) and jasmonic acid (GO:2000022) were found. In these hormone-related categories, several genes belonging to the WRKY transcription factor family such as *WRKY80* (Solyc03g095770), *WRKY81* (Solyc09g015770) and *WRKY40* (Solyc06g068460) were found up-regulated. The WRKY family proteins are important actors in the regulation of transcriptional reprogramming associated with plant stress responses (Chen et al., 2012). At PFB stage, GO terms corresponding to metabolic process (GO:0019222) were found enriched among the 57 commonly up-regulated DEGs and include genes related to the redox pathway such as glutaredoxin (Solyc01g067460) encoding a small redox enzyme known to participate to abiotic stress tolerance (Wu et al., 2017). The GO enrichment of the down-regulated DEGs at both TFB and PFB stages revealed mainly photosynthesis-related genes (GO:0009765) such as Chlorophyll a-b binding proteins and oxidoreductase activity-related genes (GO:0016491) including peroxidase, indicating that both stages are affected in photosynthesis during HS. Altogether these results suggested that the common response to HS in the five cultivars mainly corresponds to the upregulation of HS response genes, oxidative stress and hormonal pathways and the down-regulation of photosynthesis related genes.

### 3.5 Expression of reproductive development related genes is altered under HS

**Figure 7:**
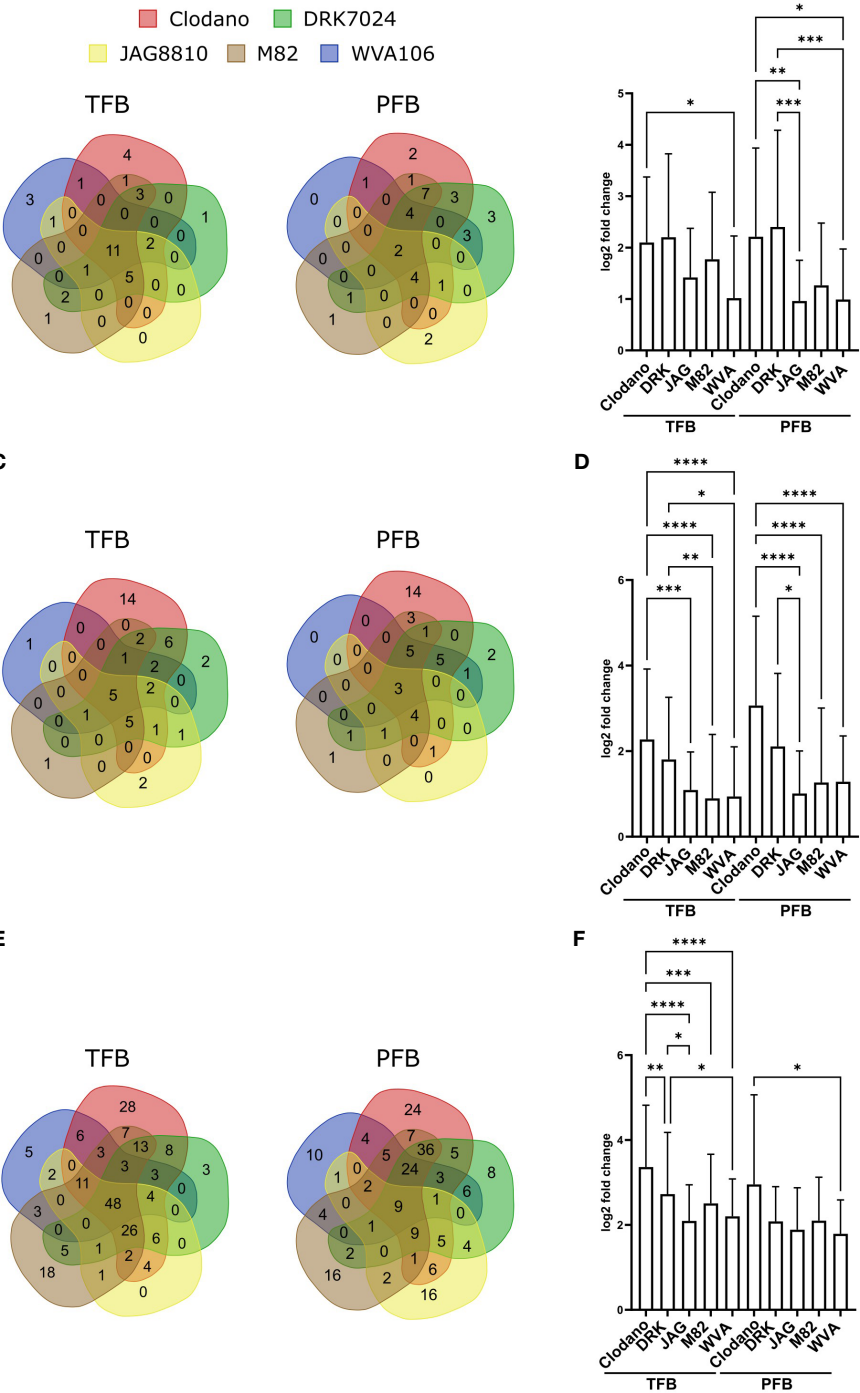
Reproductive process-, HS- and ROS-related genes response to HS between cultivars. **(A, C)** Venn diagrams presenting the overlap of up-regulated reproductive process **(A)** HS-**(C)** and ROS-**(E)** related genes between all cultivars. **(B, D, F)** Mean log2-fold change of up-regulated reproductive process- **(B)**, HS-**(D)** and ROS-**(F)** related genes between all cultivars in TFB and PFB. p-value *<0,05; **<0,01; ***<0,001; ****<0,0001 (Kruskal Wallis test).

The “regulation of reproductive process” (GO:2000241; 44 genes) and “developmental process involved in reproduction” (GO:000300; 130 genes) categories were found enriched in the pool of up-regulated and down-regulated DEGs, respectively, for all cultivars (**Table S8**) confirming that HS affected flower development. In the upregulated ones, we found genes related to flower development and inflorescence architecture such as *FRUITFULL-like MADS-box 1* (Solyc06g069430) previously described as a regulator of flowering time and inflorescence architecture in tomato (Jiang et al., 2022) but these genes were found upregulated in the pollen sensitive cultivars Clodano TFB and DRK7024 and M82 PFB. In the downregulated ones, *SlPHD_MS1* (Solyc04g008420) a PHD-type transcription factor involved in pollen formation and tapetum development (Gökdemir et al., 2022) was found downregulated in the TFB of WVA106 and JAG8810 pollen tolerant cultivars only. Interestingly, more genes corresponding to “regulation of reproductive process” were found up-regulated in pollen sensitive (52 for Clodano; 53 for DRK7024; 44 for M82) than in pollen tolerant (29 for WVA106 and JAG8810) cultivars (**Figure 7A**). In addition, the mean log2-fold change between NS and HS conditions of the common DEG belonging to this category was higher for Clodano (sensitive) than WVA106 (tolerant) in TFB and for Clodano and DRK (both sensitive) than for JAG8810 and WVA106 (both tolerant) cultivars in PFB (**Figure 7B**). This observation suggests that the pollen sensitive cultivars might be more affected in their reproductive development than pollen tolerant cultivars.

Given the fact that the applied HS affects pollen development, we further examined the transcriptional expression of several genes expressed during tomato pollen development (Chaturvedi et al., 2013) under NS and HS conditions. We also included in our dataset genes proposed to be related to tomato pollen- and tapetum development (Jeong et al., 2014; Liu et al., 2019). Few genes specifically expressed during anther development and in the microspore were found to be down-regulated under HS in several cultivars (**Table S9**). However, no clear specific response could distinguish the pollen sensitive versus pollen tolerant cultivars.

### 3.6 Expression of *HSPs* and *HSFs* genes is altered under HS

Among the genes up- and down-regulated, we identified a high number of *HSPs* (heat shock proteins) encoding chaperone proteins and *HSFs* (heat shock factors) encoding DNA-binding proteins, known to be HS-related genes. Forty-eight and 44 out of 208 tomato HS-related genes (Keller et al., 2018) were found up-regulated in at least one cultivar in TFB and PFB, respectively, for a total number of 50 genes at both stages (**Table S10**). Interestingly, more HS-related genes were found upregulated in pollen sensitive (45 for Clodano; 33 for DRK7024; 22 for M82) than in pollen tolerant (20 for WVA106 and 20 for JAG8810) cultivars and only few were common between all five cultivars suggesting a specific response of each cultivar (**Figure 7C**). Moreover, for the up-regulated HS-related genes in all cultivars, despite a similar absolute expression level under NS conditions, the mean log2-fold change between NS and HS conditions in pollen sensitive cultivars was higher than in pollen tolerant cultivars for the two stages analyzed (**Figure 7D**) suggesting that the sensitive cultivars might sense the stress in a stronger manner.

### 3.7 Expression of ROS-related genes is altered under HS

Out of 1860 DEGs, a total of 259 genes related to the response to oxygen-containing compound GO (GO:1901700) were up-regulated at both stages in all cultivars (**Table S11**). Forty-eight out of 210 in TFB and only 9 out of 212 were common between all cultivars suggesting a specific response of each cultivar in terms of ROS related genes (**Figure 7E**). Moreover, as for HS related genes, the mean log2-fold change between NS and HS conditions in pollen sensitive cultivars was higher than in pollen tolerant cultivars for the two stages analyzed (**Figure 7F**). Genes encoding ROS scavenging enzymes such as the Ascorbate peroxidase (Solyc09g007270), was found upregulated in HS condition. Antioxidant enzymes such as catalases (Solyc04g082460 and Solyc12g094620) were upregulated under HS and Solyc12g094620 displayed higher expression levels under NS condition in pollen tolerant cultivars compared to pollen sensitive cultivars in both TFB and PFB stages (between 2.1- and 2.6-fold increase in TFB and between 1- and 2-fold in PFB). Moreover, enzymes involved in ROS detoxification such as the iron superoxide dismutase (Solyc06g048410) were found up-regulated.

## 4 Discussion

### 4.1 Various tomato cultivars respond unequally to HS

The effects of different types of stresses, including HS, on flower development have been studied for a long time. It is known that the optimal temperature for growing tomato plants is 25°C during the day and 20°C at night (Alsamir et al., 2017). For sensitive tomato cultivars, if the temperatures exceed 26°C during the day and 20°C during the night, floral development is altered, with, in particular, a reduction in pollen production, viability and germination capacity of pollen grains, leading to impaired fruit set and to lower number of seeds per fruit, and, therefore, reduced fruit yield (Firon et al., 2006). Because the response to stress varies widely between genotypes (Gonzalo et al., 2021), gaining deeper knowledge of the developmental and molecular response of various cultivars may provide clues for innovative breeding strategies aiming at improving commercial tomato cultivars for better adaptability to climate change. In the present work, we investigated the responses to HS in eleven tomato cultivars producing fruits of different sizes and shapes. Observation indicates that one of the most characteristic effects of HS on floral morphology is stigma exertion, which negatively impacts self-pollination due to the excessive elongation of the styles minimizing pollen access to the stigmas and reducing fertilization, and consequently decreasing fruit yield and fruit quality (Fernandez-Muñoz and Cuartero, 1991; Giorno et al., 2013). A similar response was previously described in the tomato cultivar Micro-Tom (Pan et al., 2019) and the wild species *S. pimpinellifolium*, *S. pennellii* and *S. chilense* (Alsamir et al., 2017) as a consequence of increased temperature. Interestingly, we only observed this effect in the cultivar Sassari. The absence of stigma exertion in the other cultivars could be explained by the milder stress applied in our study compared to the harsh heat condition used in previous studies 35°C/30°C and 16 h/8 h day/night for 12 days in Pan et al. (2019) and two months in a tunnel house with a maximum of 50°C and a minimum of 30°C in Alsamir et al. (2017). The pistil exertion in the Sassari cultivar could thus indicate that this cultivar is very sensitive to HS. This high sensitivity is also highlighted by the absence of pollen germination under the HS conditions we applied (**Figure 2**). As previously observed (Giorno et al., 2013), HS triggered additional morphological alterations including curled petals and sepals, and early opening of the sepals and petals depending on the cultivar. The morphological observations after HS thus reveal that the cultivars respond differently to the stress applied.

### 4.2 HS affects pollen germination in all cultivars but with different ranges

Pollen is easily damaged by exposure to HS (Bita et al., 2011; Paupière et al., 2017; Rieu et al., 2017). The exposure of tomato plants to high temperatures has three effects on pollen grains: a reduction in the number of grains and a reduction in germination and viability (Pressman et al., 2002). The impact of HS on pollen germination was described previously in many species such as in rice (Coast et al., 2016), sorghum (Sunoj et al., 2017), peanuts (Kakani et al., 2002), spring wheat (Bheemanahalli et al., 2019) and tomato (Sherzod et al., 2020). Thermotolerant tomato cultivars have been defined as plants with higher yield, high quantity of viable pollen grains, and higher germination capacity under HS conditions when compared to thermosensitive plants (Firon et al., 2006). In our study, we found that two cultivars were possibly thermotolerant, namely WVA106 and JAG8810, since they maintain high pollen germination under HS. However, these two cultivars differ greatly with regard to their germination capacity under NS conditions (82.7% and 23.4%, respectively). Such a variability between tomato cultivars has already been reported and can be attributed to differences of pollen germination capacity between genotypes (Gentile et al., 1971; Huner and VAN Huystee, 1982; Abdul-Baki and Stommel, 1995). These variations were also observed in other studies as in spring wheat (*Triticum aestivum*) where pollen germination of 22 cultivars was compared and a germination percentages of 87% maximum and 30% minimum were observed (Bheemanahalli et al., 2019). The decrease in the capacity of pollen to germinate under HS conditions is probably due to damages in the physical structure of pollen, pollen wall composition or lower energy (sugar) status (Jiang et al., 2015; Sita et al., 2017; Sunoj et al., 2017; Djanaguiraman et al., 2018; Jagadish et al., 2021). JAG8810 might be more thermotolerant than WVA106 since the decrease of pollen germination was less important than for WVA106. In other cultivars, we found that pollen germination was lower than 10%, a very low rate probably leading to reduced seed set and yield, parameters we could not evaluate since we sampled the floral buds. These findings indicate that these 9 cultivars are sensitive to the stress applied when considering the pollen germination trait. For that trait too, it seems that the genetic background influences differently the response to HS.

### 4.3 HS affects pollen development in the sensitive cultivars

Pollen grains are formed inside the anthers through a series of developmental steps from microsporocyte meiosis to pollen release (Gómez et al., 2015). In general, pollen development is synchronized until microsporocyte meiosis I, and sometimes until meiosis II (Brukhin et al., 2003). Cell division synchronization is necessary for successful and coordinated pollination and fertilization (Magnard et al., 2001). Specifically, an environmental stress (extreme temperature or drought) induces asynchrony during pollen development and gives rise to meiotic abnormalities (Pécrix et al., 2011; De Storme and Geelen, 2020). When cultivated under continuous mild heat, a simultaneous reduction in pollen viability and appearance of anthers with pistil-like structures was observed in tomato indicating that HS impairs anther development (Müller et al., 2016). In our study, we show that pollen development is affected at very early stages by HS, impairing meiosis and leading to a delay or a desynchronization of pollen development depending on the cultivar considered.

### 4.4 Global transcriptome responses to a chronic heat stress in the five tomato cultivars

Anther and pollen development are the major flower parts being sensitive to HS (Thakur et al., 2010; Zinn et al., 2010; De Storme and Geelen, 2014), but sepals have been shown to play important roles in protecting inner floral organs from various stresses including HS (Chen et al., 2019) showing that HS affects the whole floral bud. Under the chronic heat stress we applied, not only pollen development and germination were affected in the eleven tomato cultivars, but also floral bud development and morphology were too (**Figure 1**). In order to further explore the gene expression changes in the floral buds of different tomato cultivars under HS, we performed a comparative RNAseq analysis on entire floral buds to reveal the differential gene expression in floral buds at tetrad stage (tetrad floral bud, TFB) and pollen mature stage (pollen floral bud, PFB) in five cultivars with contrasted pollen development tolerance to the HS. The transcriptome analysis revealed that similar stress-related processes were differentially regulated despite the phenotypic differences between the cultivars. In total, 244 and 126 genes were found differentially expressed similarly in all five cultivars under HS at TFB and PFB stages, respectively. In addition, the global transcriptome response at both TFB and PFB stages was largely common. This common response to HS mainly corresponded to the upregulation of HS responsive genes, oxidative stress and hormonal pathways and the down-regulation of photosynthesis related genes. The existence of such a robust consensus molecular response over different genetic backgrounds has already been described to mild drought stress in developing Arabidopsis leaves (Clauw et al., 2015). These results not only strongly suggest that pollen tolerance to HS is linked to the ability of the plant to mitigate the HS effects through a higher expression of heat-response factors, but also by activating a common response for short term extreme HS and chronic HS exposure to moderate high temperatures.

### 4.5 Reproductive development related genes under chronic HS

In our dataset, we found genes corresponding to the regulation of reproductive process being enriched in the differentially expressed genes under HS in all cultivars. These genes showed a higher level of upregulation in pollen sensitive compared to pollen tolerant cultivars suggesting that this process is more affected in the pollen sensitive cultivars. By comparing our dataset with publicly available transcriptomics data describing genes related to tomato pollen- and tapetum development (Jeong et al., 2014; Liu et al., 2019), we found a few genes specifically expressed during anther development and in the microspore, being down-regulated under HS. For example, the strictosidine synthase gene (*SlSTR1, Solyc03g053130*), a stamen-specific gene triggering male-sterility when knocked-out (Du et al., 2020), was downregulated in all cultivars at TFB stage, suggesting that the decrease in its expression under HS could be related with altered anther development. However, we could not find a clear specific response distinguishing pollen sensitive versus pollen tolerant cultivars. A possible explanation is that, since we used the entire floral bud being a highly complex structure, our RNA-seq results highlighted the global response of floral buds to the HS, hiding the specific response of the pollen and other reproductive cell types. New technologies such as spatial transcriptomics or single-cell RNAseq could be used to reveal the specific response of the different cell types making the floral buds.

### 4.6 HSFs and HSPs related genes under chronic HS

Under a short HS treatment, the *HSP* and *HSF* genes are strongly induced and play important roles to protect the plant. HSPs act by maintaining the homeostasis of the plant as they have a chaperone activity to limit the misfolding of proteins, but also to protect protein complexes like photosystem II (Vierling, 1991; Tsvetkova et al., 2002; Waters, 2013). Their expression is mainly controlled by the HS transcription factors HSFs. Our global transcriptome analysis showed that all cultivars respond to a chronic HS by maintaining an increased expression of numerous *HSF* and *HSP* genes and a stronger upregulation in sensitive- compared to tolerant cultivars. Several of the commonly up-regulated genes found in our transcriptome have been already described as important factors for HS tolerance in male reproductive tissues and, particularly, at the tetrad stage such as the HsfA2 and HsfA6b (Fragkostefanakis et al., 2015). Additionally, several HSPs (Solyc02g088610, Solyc03g007890, Solyc03g113930, Solyc03g115230, Solyc11g066100, Solyc11g071830), up-regulated in one or multiple cultivars under HS, were previously identified as belonging to heat response QTLs in small-fruited tomato population suggesting that they play a general role in HS response in many tomato cultivars (Bineau et al., 2021). Therefore, our results fall in line with previous observations regarding changes in HSPs and HSFs expression under HS, and suggest that depending on the cultivar considered, the level and diversity of HS related proteins induced may vary, potentially resulting in the different responses observed.

### 4.7 ROS- and hormone-related genes under chronic HS

The ability of a plant organ to either prevent or repair HS-induced oxidative damage is an important component of thermotolerance. This ability is mainly mediated by the induction of ROS scavengers, such as APX, under HS conditions (Suzuki and Mittler, 2006). In the present study, we found 259 DEGs related to the response to ROS including antioxidant and ROS detoxification enzymes (ascorbate peroxidase, catalases and superoxide dismutase) induced in response to HS in floral buds of all cultivars at both developmental stages. For example, the ascorbate peroxidase gene (*Solyc09g007270*) was found up-regulated in several cultivars at both PFB and TFB stages. This gene has already been reported to be responding to HS in tomato microspores (Frank et al., 2009) and could contribute to pollen development thermotolerance in tomato.

Plant hormones signaling pathways, such as abscisic acid, ethylene, salicylic acid and jasmonic acid were proposed to play an important role in plant thermotolerance (Kotak et al., 2007). In particular, salicylic acid has previously been shown to be important for anther and pollen thermotolerance in tomato (Jansma et al., 2022). In our dataset, GO related to hormonal responses including abscisic acid, salicylic acid and jasmonic acid were found enriched indicating that these pathways are affected by the HS in tomato floral buds and the underlying genes could be interesting targets for HS tolerance. The WRKY transcription factor family is regulated by hormonal signaling and plays important roles in plant stress response including HS (Cheng et al., 2021). In pepper, *CaWRKY40* has been associated with HS tolerance and its expression is regulated by SA, JA and ethylene (Yang et al., 2022). In tomato, another member of the WRKY family, SlWRKY3 has been demonstrated to be a positive regulator of thermotolerance that activates the expression of a range of HS responsive genes involved in ROS scavenging (Wang et al., 2023). The *WRKY* genes identified as up-regulated under HS in our analysis could contribute to HS tolerance and are good candidates for future functional validation studies.

### 4.8 Conclusion

**Figure 8:**
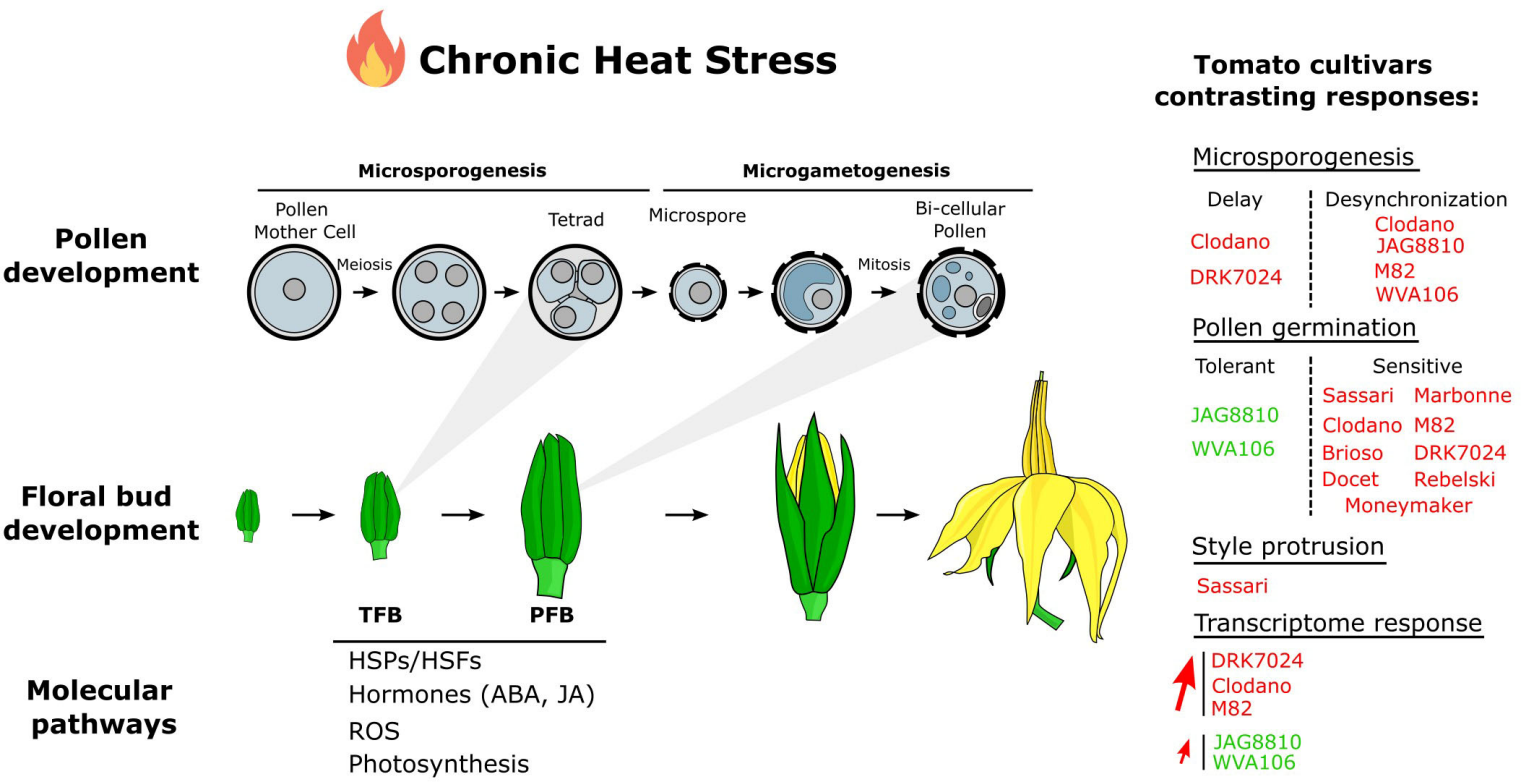
Model of tomato floral bud responses to a chronic HS.

Our results showed that chronic HS is detrimental for pollen development in all tested tomato cultivars but the tolerance to HS widely varies between genotypes (**Figure 8**). The effects of global warming with the increase in heat waves magnitude and frequency on reproductive organ development thus need to be investigated using heat waves and chronic HS rather than extreme short high temperature-stress. The transcriptome dataset obtained in the present study revealed the complex response of tomato to heat and, especially the common response between five tomato cultivars. Among the up-regulated pathways, we could find a significant enrichment of GO terms related to the HS response but also to other functional categories related to photosynthesis, hormonal responses and reactive oxygen species demonstrating that tomato floral buds are commonly responding to HS through these pathways. Although the main HS response pathways were common in all cultivars studied, the number of differentially expressed genes and their expression level under HS was found highly variable depending on the genotype, suggesting that maintaining the level of transcript abundance could be important for HS tolerance. Investigating further this robust consensus response despite different phenotypic outputs under HS might be of great interest for agricultural applications.

## Data availability statement

The datasets presented in this study can be found in online repositories. The names of the repository/repositories and accession number(s) can be found in the article/**Supplementary Material**.

## Author contributions

NB: Conceptualization, Data curation, Formal Analysis, Investigation, Methodology, Writing – original draft, Writing – review & editing. RM-P: Data curation, Formal Analysis, Investigation, Writing – original draft, Writing – review & editing. ADa: Investigation, Writing – review & editing. EM: Data curation, Software, Writing – review & editing. MZ: Data curation, Software, Writing – review & editing. ADj: Data curation, Software, Writing – review & editing. MB: Conceptualization, Funding acquisition, Project administration, Resources, Supervision, Writing – review & editing. CC: Conceptualization, Funding acquisition, Project administration, Resources, Supervision, Writing – review & editing. FD: Conceptualization, Formal Analysis, Funding acquisition, Investigation, Methodology, Project administration, Supervision, Writing – review & editing. NG: Formal Analysis, Funding acquisition, Project administration, Supervision, Writing – original draft, Writing – review & editing. MH: Conceptualization, Formal Analysis, Funding acquisition, Investigation, Methodology, Project administration, Supervision, Writing – original draft, Writing – review & editing.
