# Supplementary figures and images for: Various tomato cultivars display contrasting morphological and molecular responses to a chronic heat stress
Source: Front Plant Sci. 2023 Oct 25;14:1278608. doi: 10.3389/fpls.2023.1278608 (PMC10642206; doi:10.3389/fpls.2023.1278608)

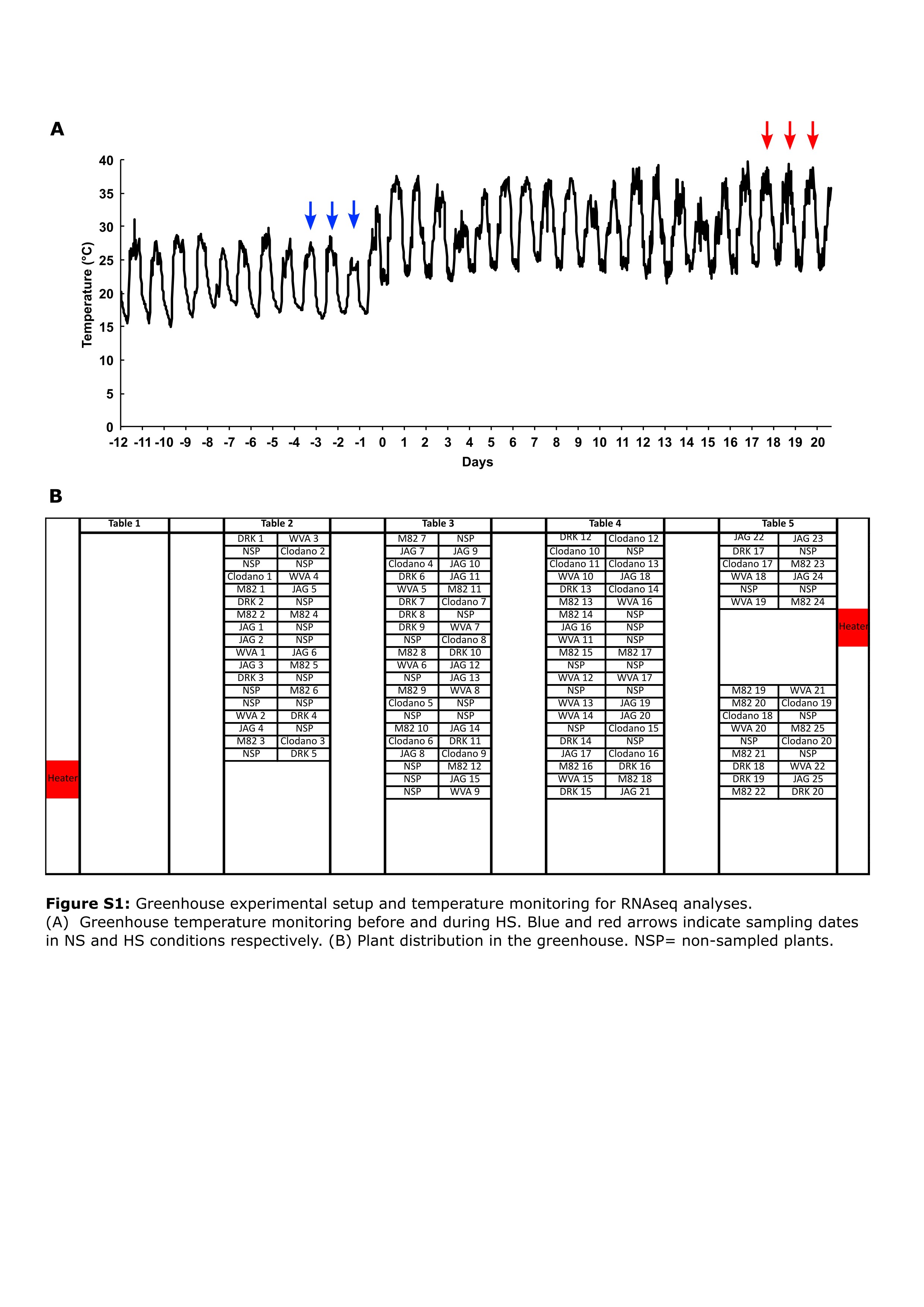

Supplement: Supplementary file 1 [file Image_1.jpeg]

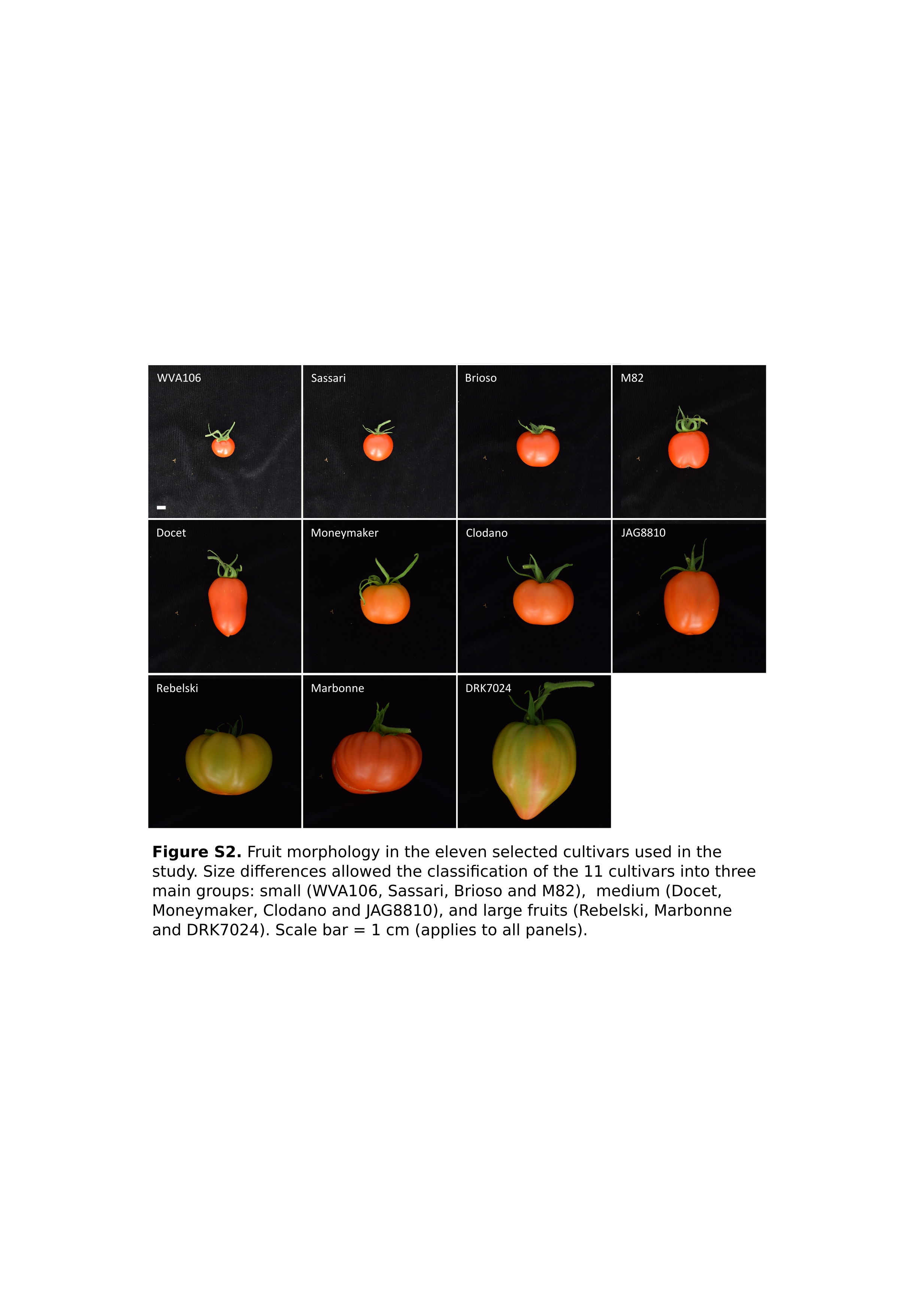

Supplement: Supplementary file 2 [file Image_2.jpeg]

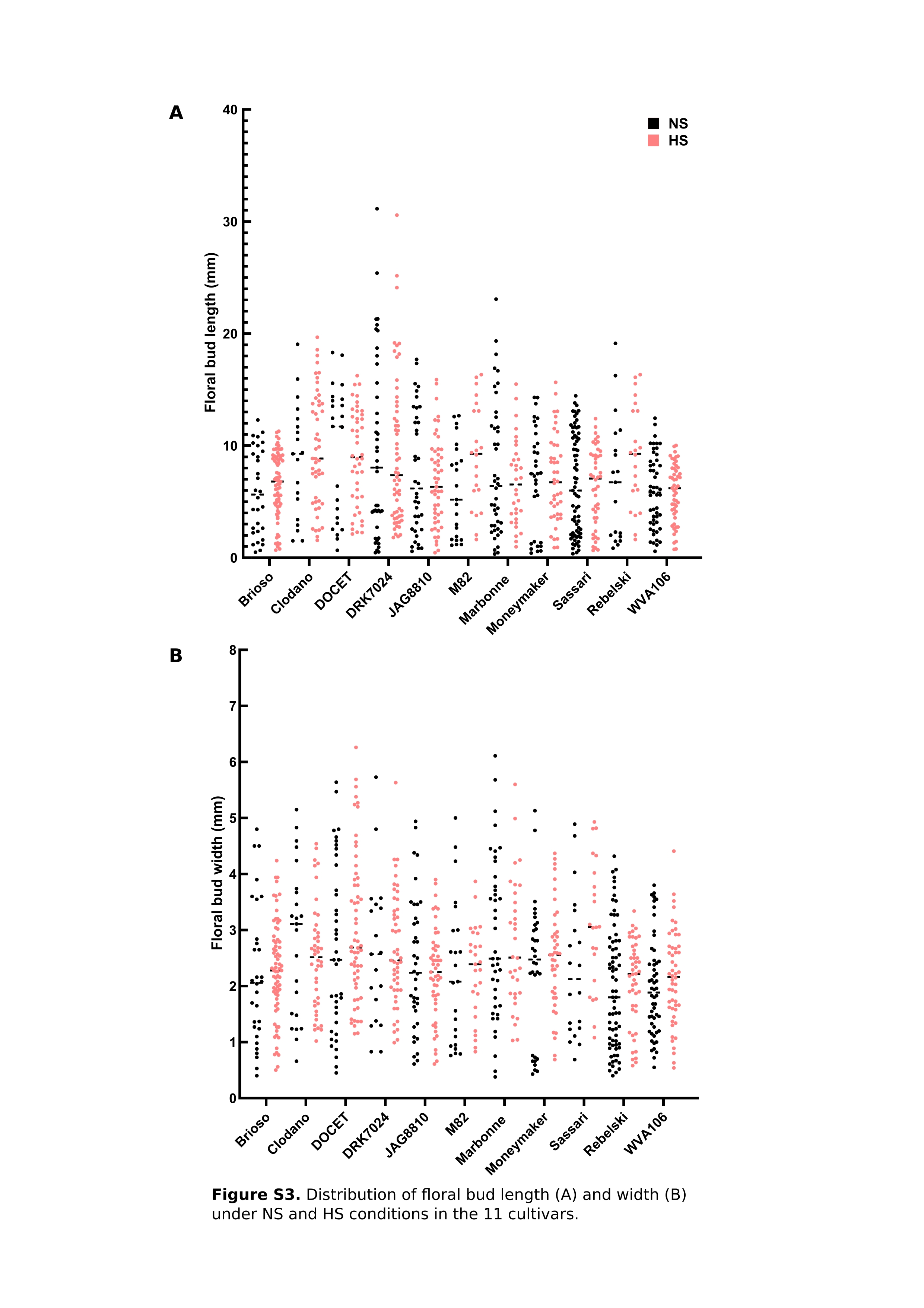

Supplement: Supplementary file 3 [file Image_3.jpeg]

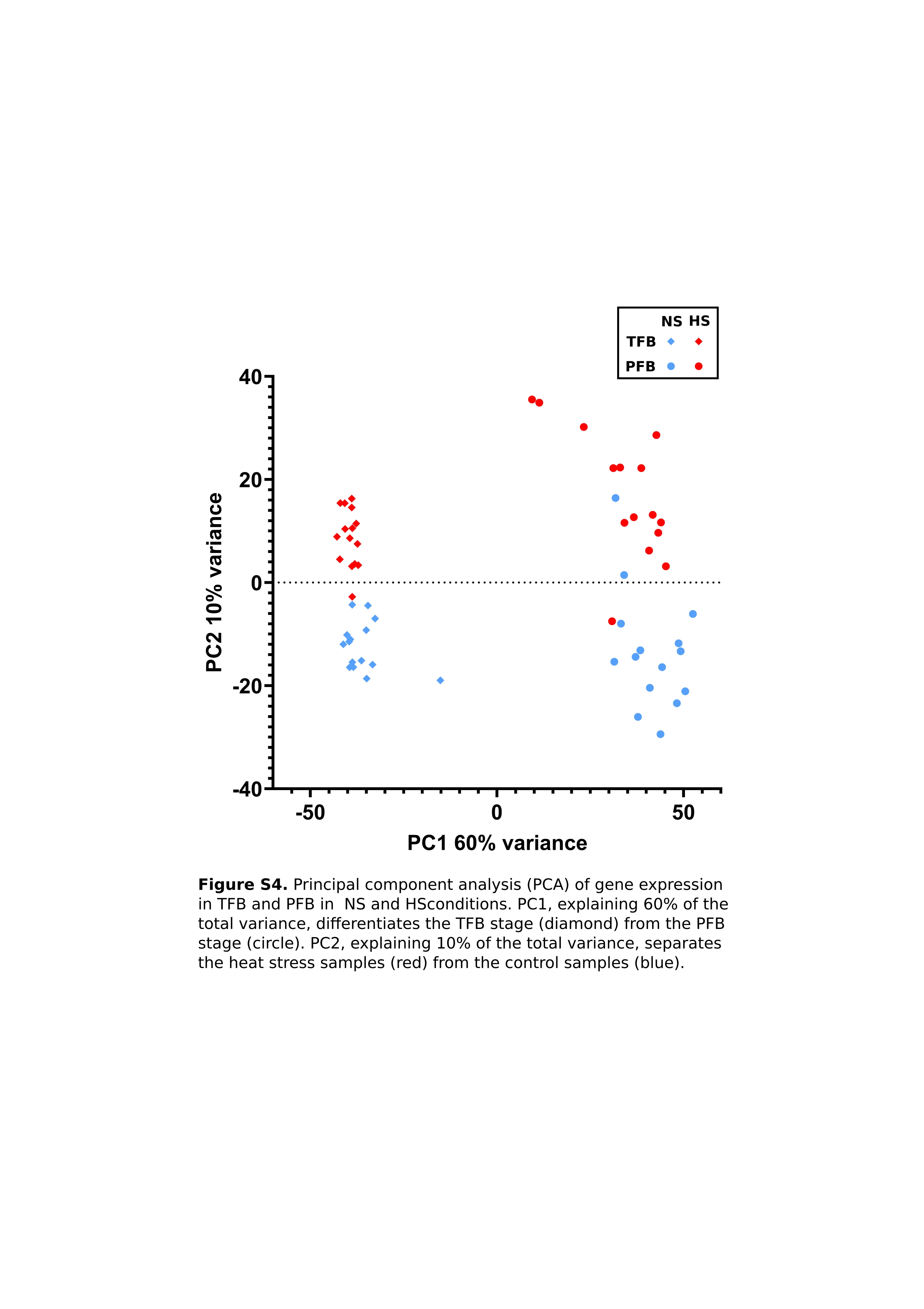

Supplement: Supplementary file 4 [file Image_4.jpeg]
